# Impact of changing diagnostic criteria for smear-positive tuberculosis: a cohort study in Malawi

**DOI:** 10.5588/ijtld.13.0811

**Published:** 2014-07

**Authors:** O. Koole, L. Munthali, B. Mhango, J. Mpunga, J. R. Glynn, A. C. Crampin

**Affiliations:** * London School of Hygiene & Tropical Medicine, London, UK; † Karonga Prevention Study, Chilumba; ‡ Ministry of Health, Karonga; § National TB Control Programme, Lilongwe, Malawi

**Keywords:** tuberculosis, microscopy, HIV

## Abstract

We assessed the impact on measured burden and outcomes of the revised World Health Organization and Malawi guidelines reclassifying people with single (including ‘scanty’) positive smears as smear-positive pulmonary tuberculosis cases. In a retrospective cohort in rural Malawi, 567 (34%) of 1670 smear-positive episodes were based on single positive smears (including 176 with scanty smears). Mortality rates and the proportion starting treatment were similar in those with two positive smears or single, non-scanty smears. Those with single scanty smears had higher mortality and a lower proportion starting treatment. The reclassification will increase the reported burden substantially, but should improve treatment access.

## RESUME

Nous avons estimé l'impact des directives révisées de l'Organisation Mondiale de la Santé et du Malawi sur la mesure du fardeau de la tuberculose (TB) et des résultats du traitement. Celles-ci reclassent les patients avec un seul frottis positif, même « faible », comme cas de TB pulmonaire à frottis positif. Dans une étude de cohorte rétrospective en zone rurale du Malawi, sur 1670 épisodes à frottis positifs, 567 (34%) étaient basés sur un seul frottis positif (dont 176 avec un frottis pauvre). Les taux de mortalité et la proportion de ceux qui ont démarré un traitement étaient similaires chez les patients avec deux frottis positifs ou un seul frottis abondant. Ceux qui avaient un frottis unique et pauvre avaient une mortalité plus élevée et une moindre proportion de mise en œuvre du traitement. Cette reclassification va sensiblement accroitre le fardeau, mais elle devrait améliorer l'accès au traitement.

## RESUMEN

En el presente estudio se evaluó la repercusión sobre la carga de morbilidad por tuberculosis (TB) y los desenlaces clínicos de las normas revisadas de la Organización Mundial de la Salud y de Malawi, que reclasifican a las personas que presentan una baciloscopia positiva única (incluidas las baciloscopias paucibacilares), como casos de TB con baciloscopia positiva. Se llevó a cabo un estudio retrospectivo de cohortes en una región rural de Malawi, de 1670 episodios de TB con baciloscopia positiva, 567 de los cuales contaban con una sola baciloscopia positiva (34%), incluidos 176 casos de una baciloscopia paucibacilar. La tasa de mortalidad y la proporción de pacientes que iniciaron el tratamiento fue equivalente en las personas con dos baciloscopias positivas y con una baciloscopia no paucibacilar. Los episodios con una sola baciloscopia paucibacilar presentaron una mortalidad más alta y una proporción más baja de iniciación del tratamiento. La nueva clasificación aumentará de manera considerable la carga de morbilidad notificada y debería mejorar el acceso al tratamiento.

IN 2007, the World Health Organization (WHO) issued new guidelines for human immunodeficiency virus (HIV) prevalent settings to increase the sensitivity and reduce the delays in the diagnosis of tuberculosis (TB) among HIV-positive patients.^1^ One positive sputum smear would become sufficient to classify a patient as a smear-positive case if the patient was known or suspected to be HIV-infected.

In 2009, the WHO recommended lowering the threshold for defining smear-positivity to one acid-fast bacillus (AFB) per smear in settings with well-functioning external quality assessment schemes, thus including smears previously denoted as scanty (<10 AFB/100 high power fields).^2^

In 2012, the National Tuberculosis Programme (NTP) in Malawi adopted these approaches, considering patients with ⩾1 smear-positive sputum sample (including single scanty smears) to be smear-positive cases. To minimise workload, two sputum specimens are now collected instead of three. Opt-out HIV testing for individuals with possible TB was also introduced.^3^

The likely impact of these policy changes on measured TB rates and the outcome in those with single positive smears are unknown. We analysed the frequency of single positive smears and the outcomes of these patients in the 15 years before the guideline changes in Karonga District, Malawi, where smear-positive TB incidence is currently ~80 per 100 000 adults per year and HIV prevalence ~8%.^4^,^5^

## METHODS

A retrospective cohort study was conducted of patients investigated for pulmonary TB from 1998 to 2012 within the Karonga Prevention Study, northern Malawi: details are given elsewhere.^4^ Project staff collect sputum from individuals with chronic cough at the hospital and peripheral clinics. In line with NTP guidelines, smears are processed in the project laboratory using fluorescence microscopy and Ziehl-Neelsen confirmation of positives. The laboratory participates in a well functioning external quality control system provided by the UK National External Quality Assessment Service. Consenting patients are counselled and tested for HIV. Patients are followed annually after completing treatment.

For research purposes, we attempted to obtain at least three sputum samples per patient at diagnosis. In the present study, we analysed data based on the first two smears taken during a 7-day period, defined as a screening episode. We differentiated single scanty and single non-scanty smears. We present the frequency of these patients and the outcomes, stratified by HIV status.

The studies were approved by the Malawi National Health Science Research Committee, Lilongwe, Malawi, and the London School of Hygiene & Tropical Medicine (London, UK) ethics committee.

## RESULTS

Between January 1998 and December 2012, 35 560 smears from 11 785 screening episodes (in 9179 individuals) were examined: 1150 submitted a single sample, 570 submitted two and 10 065 submitted ⩾3. Of these, 2426 (21%) went on to start anti-tuberculosis treatment.

Overall, including the first three smears, 1386 (12%) episodes were smear-positive, of which 331 (24%) were positive on only a single smear. Including only the first two smears gives 1335 (11%) smear-positive episodes, of which 567 (42%) were positive on only a single smear, including 176 with only a scanty smear. If the first two smears had been examined using fluorescence microscopy alone, the results would have been similar, with 1441 (12%) smear-positive episodes, of which 527 (37%) were positive on only a single smear and 168 were scanty smears.

Patient characteristics in the three groups (single non-scanty smear, single scanty smear, >1 positive) are compared in Table 1. The age and proportion that had had previous TB were similar in all groups. There was a higher proportion of women among those with single positive smears, which might be explained by the higher proportion of unknown status and HIV-positives in this group (69% vs. 56% among those with >1 positive smear) (*P* < 0.001 for both comparisons).

**Table 1:**
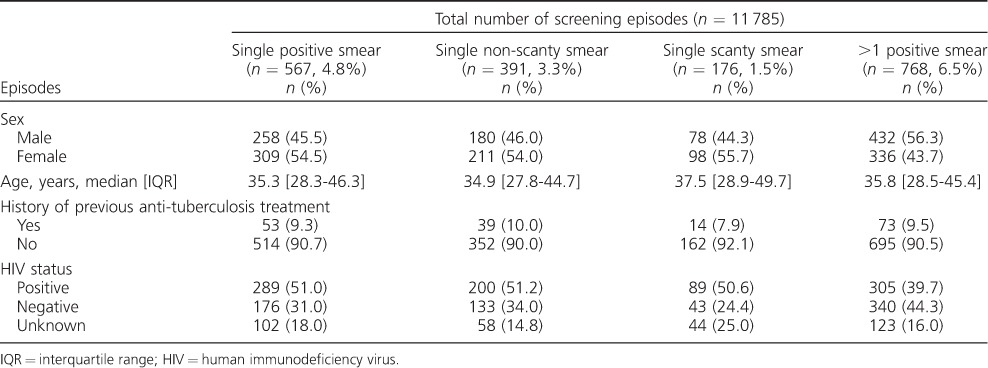
Study population and baseline characteristics of presumptive tuberculosis patients, considering results that would have been seen if only the first two smears were examined

Overall, 91 (16%) of the 567 with a single positive smear died within 1 year, compared to 101/768 (13%) with >1 positive (*P* =0.1, Table 2). Mortality was higher among those with only a single scanty smear (35/176, 20%) than those with a single non-scanty smear (56/391, 14%, *P* = 0.1). Mortality among patients with a single positive smear was higher among patients with unknown (41/102, 40%) and positive status (45/289, 16%) than among HIV-negative patients (5/176, 3%).

**Table 2.**
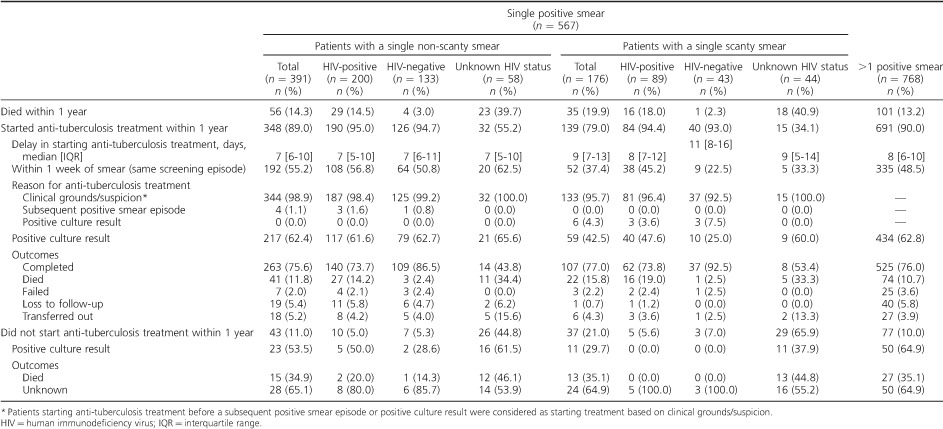
Outcomes of patients with a single positive smear and those with more than one positive smear within 1 year

The proportion starting treatment within 1 year was similar in those with a single non-scanty smear (89%) and those with >1 positive smear (90%), but lower in those with a single scanty smear (79%, *P* < 0.001). The proportions were similar among HIV-positives (274/289, 95%) and negatives (166/176, 94%), but lower in those with unknown HIV status (47/102, 46%). Only 2% of those with a single scanty smear were started on anti-tuberculosis treatment due to a later positive smear or a positive culture result; the remainder were started on clinical grounds. Of 348 patients with single non-scanty smears, 217 (62%) were culture-positive for Mycobacterium tuberculosis compared to 43% (59/139) of those with single scanty smears and 63% (434/691) of those with >1 positive smear.

Among patients with single positive smears not starting anti-tuberculosis treatment, 43% (34/80) had cultures later found to be positive; most (69%) were not HIV-tested. Of patients not started on anti-tuberculosis treatment, 35% (28/80) died (after a median of 6 days, interquartile range 3–13), including 17 who were culture-positive.

## DISCUSSION

In this study, single non-scanty and single scanty smear screening episodes were common, especially if only two sputum samples were examined. Similar results were found in Bangladesh.^6^ Reclassifying patients with scanty and single positive smears will considerably increase the number of smear-positive patients.

Similar proportions of patients with single positive smears and >1 positive smear were started on anti-tuberculosis treatment, concurring with other studies in which about 85% of patients with a positive smear were started on anti-tuberculosis treatment.^7^ However, patients with single scanty smears were less likely to start treatment and experienced higher mortality. The high mortality and lower proportions starting treatment among those with unknown HIV status probably reflects early death or loss to follow-up before HIV testing could be performed. Given the high HIV prevalence among TB patients (56% in 2006) in this setting,^8^ most of those with unknown status were probably HIV-positive. Rapid point-of-care tests such as the Xpert^®^ MTB/RIF assay (Cepheid, Sunnyvale, CA, USA) may reduce these early losses.^9^

Under current WHO and Malawi guidelines, all 567 presumptive TB patients would have been HIV-tested unless they opted out, resulting in 22% more presumptive TB patients knowing their HIV status. Based on the updated criteria for smear-positive TB, all 567 patients (+16%) would probably have been started on anti-tuberculosis treatment earlier. All HIV-positive TB patients are eligible for antiretroviral therapy treatment,^10^ which should further reduce mortality.^11^

These changes increase the sensitivity of TB diagnosis, and are likely to reduce treatment delay and mortality among a group of patients who previously had a poor prognosis. The effect on TB notification rates will be substantial; it will be important to note this classification shift when analysing trends over time.

## References

[1] World Health Organization (2006). Improving the diagnosis and treatment of smear-negative pulmonary and extra-pulmonary tuberculosis among adults and adolescents: recommendations for HIV-prevalent and resource-constrained settings.

[2] Ramsay A, Cuevas L E, Mundy C J (2009). New policies, new technologies: modelling the potential for improved smear microscopy services in Malawi. PLOS ONE.

[3] Malawi National Tuberculosis Control Programme (2012). National Tuberculosis Control Programme Manual.

[4] Crampin A C, Glynn J R, Fine P E. (2009). What has Karonga taught us? Tuberculosis studied over three decades. Int J Tuberc Lung Dis.

[5] Floyd S, Molesworth A, Dube A (2013). Underestimation of HIV prevalence in surveys when some people already know their status, and ways to reduce the bias. AIDS.

[6] Van D A, Salim A H, Cooreman E (2004). Scanty AFB smears: what's in a name?. Int J Tuberc Lung Dis.

[7] Squire S B, Belaye A K, Kashoti A (2005). ‘Lost’ smear-positive pulmonary tuberculosis cases: where are they and why did we lose them?. Int J Tuberc Lung Dis.

[8] Munthali L, Mwaungulu J N, Munthali K, Bowie C, Crampin A C. (2006). Using tuberculosis suspects to identify patients eligible for antiretroviral treatment. Int J Tuberc Lung Dis.

[9] Boehme C C, Nabeta P, Hillemann D (2010). Rapid molecular detection of tuberculosis and rifampin resistance. N Engl J Med.

[10] World Health Organization (2010). Antiretroviral therapy for HIV infection in adults and adolescents: recommendations for a public health approach. Revision 2010.

[11] Blanc F X, Sok T, Laureillard D (2011). Earlier versus later start of antiretroviral therapy in HIV-infected adults with tuberculosis. N Engl J Med.

